# Developing and evaluating interventions that are applicable and relevant to inpatients and those who care for them; a multiphase, pragmatic action research approach

**DOI:** 10.1186/1471-2288-14-98

**Published:** 2014-08-18

**Authors:** Jack J Bell, Tony Rossi, Judith D Bauer, Sandra Capra

**Affiliations:** 1 Department of Nutrition and Dietetics, The Prince Charles Hospital, Rode Road, Chermside, Brisbane 4035, Australia; 2 Centre for Dietetics Research, School of Human Movement Studies, The University of Queensland, St Lucia, Brisbane 4072, Australia; 3 Australian Centre of Sport, Physical and Health Education Research, School of Human Movement Studies, The University of Queensland Brisbane, Brisbane 4072, Australia

**Keywords:** Pragmatic, Action research, Malnutrition, Aged, Hip fracture, Qualitative research

## Abstract

**Background:**

Randomised controlled trials may be of limited use to evaluate the multidisciplinary and multimodal interventions required to effectively treat complex patients in routine clinical practice; pragmatic action research approaches may provide a suitable alternative.

**Methods:**

A multiphase, pragmatic, action research based approach was developed to identify and overcome barriers to nutritional care in patients admitted to a metropolitan hospital hip-fracture unit.

**Results:**

Four sequential action research cycles built upon baseline data including 614 acute hip-fracture inpatients and 30 purposefully sampled clinicians. Reports from Phase I identified barriers to nutrition screening and assessment. Phase II reported post-fracture protein-energy intakes and intake barriers. Phase III built on earlier results; an explanatory mixed-methods study expanded and explored additional barriers and facilitators to nutritional care. Subsequent changes to routine clinical practice were developed and implemented by the treating team between Phase III and IV. These were implemented as a new multidisciplinary, multimodal nutritional model of care. A quasi-experimental controlled, ‘before-and-after’ study was then used to compare the new model of care with an individualised nutritional care model. Engagement of the multidisciplinary team in a multiphase, pragmatic action research intervention doubled energy and protein intakes, tripled return home discharge rates, and effected a 75% reduction in nutritional deterioration during admission in a reflective cohort of hip-fracture inpatients.

**Conclusions:**

This approach allowed research to be conducted as part of routine clinical practice, captured a more representative patient cohort than previously reported studies, and facilitated exploration of barriers and engagement of the multidisciplinary healthcare workers to identify and implement practical solutions. This study demonstrates substantially different findings to those previously reported, and is the first to demonstrate that multidisciplinary, multimodal nutrition care reduces intake barriers, delivers a higher proportional increase in protein and energy intake compared with baseline than other published intervention studies, and improves patient outcomes when compared with individualised nutrition care. The findings are considered highly relevant to clinical practice and have high translation validity. The authors strongly encourage the development of similar study designs to investigate complex health problems in elderly, multi-morbid patient populations as a way to evaluate and change clinical practice.

## Background

Within the context of the ever-burgeoning expectation that medical research becomes translational, that is, that it informs ‘real’ practice in ‘real’ medical settings (clinics, hospitals and other facilities), the search for ‘practices’ that ‘work’ is serious. Of course this quest can be hampered by the inexorable pursuit of demonstrable clinical effects of interventions founded upon gold standard methods. This invariably means that clinical effects demonstrated through the rigours of randomised controlled trials (RCTs) maintain a mesmerizing grip on the definitions and descriptors of what stands for ‘high quality’ research and the often-lofty claims to truth. And yet when it comes to, for example, nutritional RCTs and reviews of RCTs investigating malnutrition in acutely unwell, multi-morbid elderly inpatients, (in this case hip fracture), the relevancy and applicability of findings to patients and those who treat them are often found wanting. Limited understanding and application of malnutrition screening and diagnostic measures by those with limited or misdirected nutritional knowledge have led to the under-diagnosis or misdiagnosis of malnutrition in highly cited RCTs and systematic reviews of these [1]. Nutrition intervention studies in hip fracture routinely report highly constrained research environments in order to demonstrate clinical effect of highly controlled, often one-dimensional interventions in the presence of these confounders, despite the recognised need for these patients to receive ‘comprehensive’ (that is, multidisciplinary and multimodal) care [2]. Further to this, this predisposition of patients to multiple comorbidities and complications dictates the exclusion of those perhaps most likely to benefit from interventions in order to clearly demonstrate cause and effect in the purest of senses. A review of RCTs in any select elderly inpatient population prone to comorbidity regardless of disease or intervention type will invariably demonstrate substantial selection (or recruitment) bias; and at least in the case of nutritional studies, the recruitment of younger, and more generally homogenous less morbid patients may mask the effect of nutritional interventions on outcomes. It is therefore not surprising that RCTs and reviews of these have failed to clearly define consistent and adequate evidence to guide bedside nutritional care in patients with acute hip fracture [1].

This is another significant concern when it comes to patient care. RCTs clearly provide the bulk of ‘evidence’ for evidence based medicine or EBM (see later where we distinguish this term from evidence based practice or EBP). Williams and Garner [3] suggest that EBM has become a stick by which clinicians are beaten by policy makers (they also implicate academics here). Hotopf [4] is more withering in his criticism arguing that RCTs are more often than not designed to address either the wrong question or questions so narrow that the solutions provided are of minimal help to the clinician thereby limiting capacity for quality care and appropriate treatment. Though Hotopf is not in favour of abandoning RCTs altogether, he does argue that they need to be expanded to be of any practical use [4]. His suggestion is that RCTs be expanded along pragmatic lines. We discuss the value of pragmatic trials and their relationship with action research (AR) later, but are quick to note that both RCTs and pragmatically focused studies have a rightful place within the spectrum of research. For now however, suffice it to say it appears that the time is right for clinical interventions to be informed by a broader church. To this end, this paper will present a case for AR as both intervention *and* research method. Not only is this consistent with the purer purposes of AR (social change and improvement of practice), it also offers a way forward for medical researchers and practitioners to be more closely aligned in the pursuit of improved quality of health care. As part of the case we advance here, we provide evidence of such an approach undertaken in a hospital based clinical environment specializing in the treatment of hip fracture.

### Some history and the central tenets of AR

Later we include a detailed account of how AR was used in this study. For now however a potted AR history or background seems appropriate, especially as this history articulates with the broader social concerns in addition to the clinical realities of modern health care. Detailed accounts of the structural procedures complete with diagrammatic representations of the cyclical nature of the AR process are ample [5-7]. This paper is more about how AR can be regarded as a legitimate clinical intervention and how it may be usefully applied in the broadest translational sense.

We begin by providing some antecedents from the early beginnings and original intentions of AR to its more recent manifestations in health-based research. This brief chronicle places the root of AR as a democratic form of social practice and alludes to its shift to a technical process of improving professional practice with little or no social intent. We acknowledge that this type of evaluative work serves a purpose, though it is generally regarded as a deviation from the social mission of AR. At a time when public service (including public health) is framed by a neoliberal discourse, with emphasis on the individual, competition, and market driven policy, we are concerned that AR could be narrowly used as a simple analytical or even benignly reflective tool. However we would argue that in clinical and medical settings not only is this inadequate, it would squander the opportunity AR offers to truly observe, improve, and evaluate routine clinical practice as it happens with the capacity to bring about change through the cyclical protocol; and further, to engage both patients and those involved in their care in this process.

Though some trace the origins of AR back to John Dewey’s work in the 1930s [8], this tends to focus more what Schön (1983) might call reflection-on-action [9]. This epistemological lineage is not insignificant; however Kurt Lewin is generally credited with coining the term action research [10]. He came to prominence in the 1940s for his work, which attempted to bring together “the experimental approach of social science with programs of social action in response to major social issues of the day” (p.29) [11]. In particular, Lewin saw AR as having great potential to improve the position and wellbeing of minority groups in post-war America, and his work within ‘race relations’, (a term that must seem somewhat anachronistic), across the country is a testament to his commitment to social betterment [10]. His skill in setting up projects that involved deep levels of consultation through the now well-recognised steps and spiraled progressions of AR was a major achievement of the time and universally acknowledged today. He was well recognised for work with groups where, through a ‘planning – fact-finding – execution’ sequence, behaviour changes could be brought about that would advantage the group whether it was food consumption practices or group solidarity and factory worker rights in an increasingly industrialized and urbanized America. The connection of this history to health care may not be immediately obvious. However, malnourished elderly hip fracture inpatients may be considered as stranded skeletons hiding in hospital closets quietly waiting to be rescued [12]. In other words this group tends to be somewhat marginalized within the context of health care and to that end, the applicability of Lewin’s work to this field appears justified.

There is no doubt that this democratized approach to social inquiry as advocated by Lewin and others around the time challenged the scientific orthodoxy of the day, and in many respects in spite of its broader acceptance in contemporary research circles, it probably still does.

### AR in medicine, health and clinical practice

It is not difficult to see how AR might apply to patient care in clinical settings. As Vallenga et al. suggest AR is “suitable because it is a form of research enabling practitioners and consumers to participate in the development of knowledge which they themselves will subsequently use or will be used in their care” (p.81) [8]. In other words the conditions of and for clinical care are best observed, developed, implemented, refined and evaluated through the collaborative and cyclical reflective structures shared between practitioner teams and the patient. What is especially important about Vallenga et al’s position here is that it frames the idea of ‘practice’ in a particular way. Practice as it is being used here is very much about the co-construction of knowledge that can be recruited to affect changes to care. In other words it is ‘care’ that is the practice, not the technical components of the procedures to bring it out. This approach clearly brings into focus the patient and further highlights the need for those who care for them to dynamically work together across systems, processes, and interventions to procure effective multidisciplinary and multimodal care. So as McTaggart describes, practice is not some automated procedure, repetitive task, or the implementation of a technique (or even policy for that matter) [13]. Hence when we talk about AR as a way to improve practice we would caution being lulled into a liberal discourse of autonomy, which tends to undermine any sense of responsibility. Habermas has a term that perhaps is more fitting [14]. He talks of ‘mature autonomy’. As McTaggart points out, this co-joining of autonomy and responsibility fits more closely with the idea of practice as a social endeavor and in the case of this study, that responsibility was both to and with patients as a way of improving and reporting quality care within the constraints of routine clinical practice [13]. As McIntosh suggests this approach to the improvement of practice requires a shift from evidence-based medicine that invariably establishes a series of routines and interventions based on clinical trials to evidence based practice that is better informed by pragmatic trials [15]. At the same time McIntyre would argue that this distinction between evidence based medicine and evidence base practice is an example of how the idea of practice should be separated from the idea of institutions [16]. In the case of this study we could argue that medicine (or indeed health) might be perceived as an institution (given its power this is not too much of a stretch of the imagination). Care, on the other hand, is the practice that goes on before, within, and beyond the context of that institution (be it in a hospital clinic or health centre). We explore this relationship later in the paper.

### A vehicle for delivering relevant, applicable, and measurable healthcare improvements

As Whitehead, Taket & Smith suggest, AR is increasingly regarded as not only a legitimate approach to research in health and medical settings, but also as a particularly effective process for supporting organisational change [17]. Within clinical settings changes to organisational practice are the key to improved health care. Indeed as we have already suggested, clinical or randomised trials can be somewhat insensitive to the nuances of service delivery in health where clinical significance is of far greater importance than statistical significance where the outcomes are patient related and change is demonstrable; no matter how small or large and predominantly based on the clinicians knowledge of the patient [18]. However as Whitehead, Taket & Smith continue, AR has been slow to catch on in the context of heath research, probably for the reasons of perceived weakness to which we alluded to earlier [17]. This poses a serious problem since as Meyer suggests, “barriers to the uptake of the findings of traditional quantitative biomedical research in clinical practice are increasingly being recognised” (p. 178) [19]. As Meyer says, the attraction of AR is that it represents a form of inquiry whereby researchers work with and for people, rather than conduct research *on* them, and in this sense it represents a form of democratized research entirely consistent with Lewin’s original premise. In other words it is the style of the research rather than its methods that are different. This also dispels the myth that AR is confined to or synonymous with qualitative research. In this project a number of data gathering techniques was used through the AR process including various numerical measures all of which contributed to a broad canvas of patient care, the changes in practice and the consequences of those changes.

### Connections to pragmatic trials

Discussions of pragmatism invariably start with the question “will a proposed intervention work in (so-called) real life?” Hence where explanatory trials measure symptoms or markers, pragmatic trials have as their focus a range of outcomes that focus on the patient’s wellbeing. In other words pragmatic trials using a range of data sources aim to work with patients rather than ‘on them’. As we have already argued, this is entirely consistent with AR. As Patsopoulos [20] points out, rather than distinguishing between explanatory and pragmatic trials (PT), it might be better to see them as points on a continuum; both indispensible but different in what they reveal. Hence the naturalistic (a word sometimes used synonymously with pragmatic – though we are cautious here) setting of a clinic provides opportunities to see what works in practice [20].

It is interesting to note that in the 2013 Australian Government Strategic Review of Health and Medical Research, pragmatic trials (much less AR!) barely warrant a mention [21]. There is an acknowledgement in the document that a broader range of research activities should be encompassed and that practitioner research should be encouraged. However, these are hardly ringing endorsements of alternative paradigms, serving to underline the somewhat tepid enthusiasm for research paradigms that sit outside of RCT convention. Baker [22] acknowledges that part of the problem is that there is “no immutable formula for successful implementation of innovations” (i30). Yet he makes a case to suggest that, (for example), case study methods are under-utilised as a way of bringing about change to care practices, and that the knowledge created through evidence based practice solutions have to be built upon the way such solutions can be implemented. In essence he is arguing for research and practice to be far more closely aligned; indeed we might even argue that they are one and the same thing. This is entirely consistent with an AR approach to change.

However, a shift in research paradigms may be emerging. Traditional models have defined RCTs as the gold standard design for evidence generation, and limited pragmatic, qualitative, prognostic or observational studies to pawns in the hierarchy of evidence [23,24]. However, clinicians, funding bodies, academics and publishers may be beginning to detect chinks in the armour of RCTs, and in response to the need to economically justify research agendas and support evidence based practice, are starting to recognise the need to also consider studies focused towards relevancy and applicability rather than overwhelmingly prioritising analyses of cause and effect under highly controlled, artificial conditions [25]. This climate change is promoting, in line with Lewin’s [10] theory, the unfreezing of accepted norms and acknowledgement that a variety of study designs focused towards the patient, the question(s) under investigation, and applicability and relevancy of the research to routine clinical practice; rather than continuing to promote highly reductionist or post-positivist focused research as the standard by which all others are judged [26]. One such illustration is an extension to the Consort Guidelines for reporting pragmatic clinical trials which provide a clear mandate for clinicians and researchers to justify pragmatically focused trials as both meaningful and relevant to patients, clinicians, and the broader healthcare community [27].

### An AR study: nutritional care in hip fracture

As an example of how pragmatically focused AR can be used in clinical settings, not only to change practice but to develop an evidence base for change, we present here a study conducted from November 2010 to September 2012. The setting is a clinical environment under change to develop best practice whilst incorporating generally understood conventions for care based on scientific trials. We allude to the results and outcomes in general terms as these have been published and presented elsewhere [28-31]. Of equal importance in our view are the processes by which care practices were developed and adjusted on the basis of the AR cycles.

## Methods

**Figure 1:**
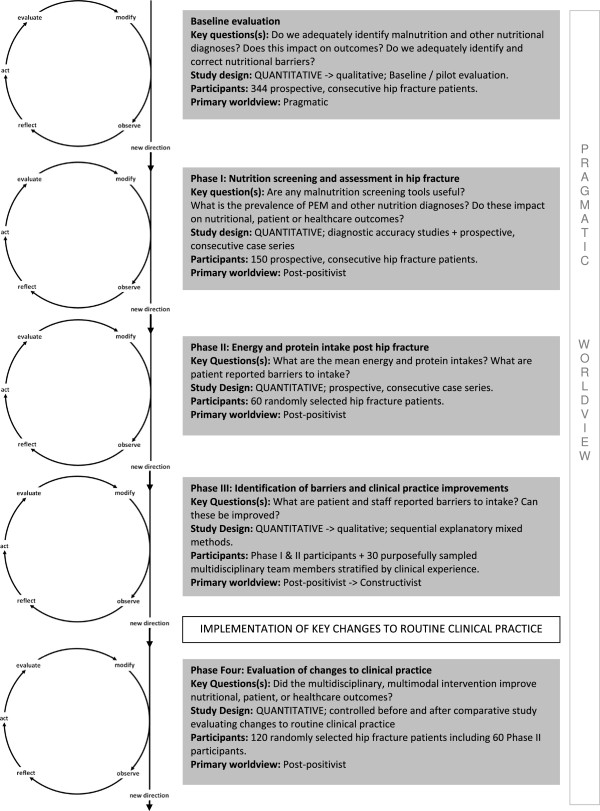
**Identifying and overcoming barriers to nutritional care in patients with acute hip fracture.** Figure 1 demonstrates the multiple phases of this action research approach. Action research cycles are based on those presented by McNiff & Whitehead [37]. Baseline evaluation to phase III represent the observational phase of an action research cycle and also provide baseline data for a before-and-after prospective interventional trial. The implementation of key changes to routine clinical practice (the ‘intervention’) are then evaluated in phase IV.

A multiphase, pragmatic, AR project with four sequential AR cycles building on baseline data was developed to identify and overcome barriers to nutrition care in hip fracture between November 2010 and September 2012 (see Figure 1). Six hundred and forty one acute hip fracture inpatients were included across all phases and 30 purposively sampled multidisciplinary clinicians actively working in the care unit were included in focus groups stratified by clinical experience as defined by positional accountability. Methods for each phase are clearly described elsewhere [28-31]; ethics approvals were obtained from the Prince Charles Hospital (HREC/11/QPCH/90; HREC12/QPCH/83) and The University of Queensland (HMS11/0607; HMS12/0904) human ethics committees. The phases can be presented according to an AR cycle as follows:

### Observe

Phase I built on baseline data and observed whether commonly applied nutrition screening and assessment processes may present as barriers to nutrition care in hip fracture and included two diagnostic accuracy studies and a prospective, consecutive case series [29,30,32,33]. Phase II reported a further prospective, consecutive case series to quantitatively investigate protein-energy intakes in post hip-fracture inpatients. This phase aimed to quantitatively report observed and reported barriers to inpatient protein-energy intake. Phase III built on earlier results including an explanatory mixed methods study to further expand and explore additional patient and clinician barriers and facilitators to nutritional care [28]. These three phases provided baseline data for comparison with post-intervention data.

### Reflect and act

Following observation of barriers and potential facilitators to nutrition in hip fracture inpatients, the multidisciplinary treating team identified potential improvements to routine clinical practice. These were trialed, adjusted as required, and then embedded into routine clinical practice as a new multidisciplinary, multimodal nutritional model of care. This was considered the ‘intervention’.

### Monitor and evaluate

A quasi-experimental before-and-after pragmatically focused study was then used to compare patient and healthcare outcomes following implementation of the new model of care with baseline data associated with the individualised nutrition care approach [31].

## Results

Baseline data evaluation suggested a patient group potentially predisposed to wasting and cachexia which was worthy of further open minded exploration across the continuum of nutrition care [33,34]. Whilst no a priori assumptions were made the treatment team was not at all surprised that the most commonly applied and recommended nutrition screening tools were highlighted as performing poorly in routine clinical practice despite previously published explanatory studies purporting their validity for this purpose; this was demonstrated to lead to substantial patient and healthcare implications [29,30]. Another diagnostic accuracy study reported that measures of malnutrition most commonly applied in nutritionally focused hip fracture RCTs were not useful for this purpose when applied in routine clinical practice [32]. Findings from this study also suggested the broad malnutrition prevalence reported across acute hip fracture studies may be attributed to differences in commonly reported nutrition diagnostic measures in addition to highly biased study population recruitment. Malnutrition was also identified as an independent predictor of poor patient and healthcare outcomes [32].

Data from Phase II highlighted that an intensive, individualised nutrition approach was unsuccessful in delivering adequate nutritional care to hip fracture patients resulting in mean protein and energy intakes less than half of estimated requirements and a substantial level of inpatient malnutrition incidence [28]. This was attributed to identification of novel and substantial barriers to hip fracture inpatient intake identified across observational phases including a higher prevalence of patient co-morbidities, baseline malnutrition, a cognitive impairment than reported elsewhere. In addition there was evidence to suggest that patients considered malnutrition and inadequate intake not to be a problem, as well as patient and clinician perceptions that treatment for malnutrition was not a priority [28]. Key facilitators to improving nutrition care in acute hip fracture patients were also identified as part of this phase.

The new multidisciplinary and multimodal nutritional care model required changes to routine clinical practice developed by the treating team in response to findings from baseline to Phase III. Key service improvements included promoting nutrition as a medicine, adopting a multidisciplinary approach to the delivery of nutritional care, increased delegation of nutrition care to be delivered by assistant staff, foodservice enhancements, and improving knowledge and awareness [31]. Following completion of phase IV, a prospective, controlled before and after comparative interventional study aligned to the CONSORT guidelines for pragmatic clinical trials was reported [27,31]. Despite a high baseline model of care, multidisciplinary nutrition care resulted in significantly and clinically relevant reduced intake barriers, doubled protein and energy intakes, a 75% reduction in nutritional deterioration, and almost tripled home discharge rates. Trends also suggested a reduction in median length of stay; inpatient mortality remained low across groups.

## Discussion

Broadly, the outcomes of this study demonstrate that the pragmatic, AR approach to nutritional care in hip fracture was important for identifying and overcoming barriers to nutrition in hip fracture across the nutrition care process. The final result demonstrated substantial improvements in related patient and healthcare outcomes, at the bedside, contrasting existing intervention studies to date that predominantly report non-significant or inconsistent nutrition related outcomes in hip fracture inpatients [1]. In our view this provides an important contribution to the literature [28-31].

Differences in explanatory versus pragmatic research attitudes across the research process, including the question, setting, participants, intervention, and outcomes, and relevancy to practice, may account for the markedly different findings reported here [27]. Tightly controlled ‘explanatory’ clinical trials are purposefully designed and ideally placed to demonstrate cause and effect (efficacy). However these frequently provide little ‘real life’ direction in developing quality care processes. As an example, studies investigating nutrition care in hip fracture have demonstrated a high likelihood of study bias, and consequently describe inclusion of younger, less comorbid, and less cognitively, functionally or physically impaired patients compared with that encountered in routine clinical practice [35,36]. Highly explanatory trials have also reported rates of malnutrition risk, malnutrition, unfavourable postoperative outcomes, and/or mortality less than that observed or expected [29,30,32,33,36]. These skewed research populations are considered likely to have diluted or negated the potential effect of interventions under investigation and have also limited the applicability of outcomes to routine clinical practice.

Highly explanatory trials in this population have also displayed limited attention to describing and/or adjusting for comorbidities and confounders, poor compliance and adherence to interventions, limitations with selection and application of outcomes measures, and/or small sample sizes and statistical methodological limitations; consequently pooled RCT data provides at best limited support for nutritional interventions post hip fracture, and the applicability and relevancy of these findings to routine clinical practice needs to be carefully considered [1,25].

As an alternative, the authors suggest that the key benefits associated with pragmatically focused AR, rather than the specific interventions themselves, should be considered as pivotal in regards to the positive outcomes observed in this study (Table 1). Pragmatic clinical trials, especially those built around the AR cycles of evaluation and reflection, aim to describe the usefulness of particular practices in routine clinical care with the capacity to change practice that does not appear to be effective in patient improvement.

**Table 1:** **Describes key benefits and challenges of pragmatic action research identified by the authors and current literature **[10,25,27,37,39]

**Benefits**	**Challenges**
Provides a systematic approach to facilitates flexible development, evaluation and publication of multimodal, multidisciplinary interventions and systematic improvements to routine clinical practice	Research paradigm impacts on outcomes and should be considered as an intervention
	Outcomes measures need to be available within the scope of routine clinical practice
Problem centric, practical, pluralistic epistemological approach placing an emphasis on the question and consequences of research rather than the research paradigm	
	Limited ability to demonstrate ‘cause and effect’
Allows and encourages research to be conducted within routine clinical practice	Limited clinician skills, understanding and application of action research, pragmatically focused trials, and/or multi-phase mixed methods research
Engages patients and clinicians to identify barriers and develop solutions and participate as co-researchers	
Harnesses skills of everyday practitioners in the absence of an additional training, resources, or environmental modifications	
	Complex nature of the design
	Difficult to define multiple phases as part of the one program
Maximises participation rate, allows participant recruitment with minimal or no selection bias, and does not emphasise the requirement for strictly controlled, limited variables, placebos or blinding	
	Require skilful connection of multiple phases or strands and the ability to transition between/across worldviews
Develops and supports multiple perspectives of reality and diversity of views rather than simplistic acceptance or rejection of a single hypothesis	
	Changes within the research team and environment need to be considered
Considerate towards investigating complex interventions that may be impacted by confounders	
Flexibly addresses interconnected research questions across a breadth of enquiry	Post-positivist attitudes focusing on the interaction between highly selected specific variables (reductionism), cause and effect (determinism), detailed variable measurement, numerical analysis and reporting (quantitative techniques)
Allows development and incremental expansion and adaptation of interventional strategies in response to feedback, resource and environment changes throughout the study period	
Prioritises relevant economic, objective, and subjective outcomes measures available for measurement in real world applications that are relevant to participants, funding bodies, healthcare providers, and the community	
	Knowledge is uncertain and outcomes are not assumed
Facilitates exploration of root causes of expected and unexpected findings	Open ended approach requires regular communication of updates and changes to clinicians and ethical bodies
Allows triangulation of results to corroborate findings	
Promotes sustainability through engagement of multidisciplinary team members	May be difficult to meet publishing requirements/formats in quantitatively focused journals
Prioritises translation validity and applicability of outcomes to routine clinical practice	
	Highlighting utility of variety of research paradigms and worldviews within and across projects rather than a ‘one size fits all’ approach
Allow reporting and publication across the course of an extended project	

AR engages practitioners in investigating, evaluating and disseminating their work; a pivotal assumption of this approach is that knowledge is uncertain and outcomes are not assumed [37,38]. Again, this is in contrast to the methods and subsequent results of RCTs in this study population which are almost universally restricted to rigorous testing of a single dimensional hypothesis formulated prior to the start of the study, whether related to oral nutritional supplementation, enteral tube feeding, or parenteral nutrition, or alternate models of care [1]. Therefore it should not be surprising that studies focused towards individual interventions, in the absence of targeted education or coordination of care strategies, report limited interventional adherence leading to inconsistent nutrition, patient and healthcare outcomes in a patient population recognised as requiring comprehensive, multidisciplinary care [1,2,36].

Results from this study further demonstrate that a multiphase, pragmatic AR approach provides a dynamic framework to encourage the incremental development and inclusion of open ended and progressive questions by clinicians as new challenges emerge [37,39]. Inclusion of multiphase mixed methods within this research framework allowed both existing and new research questions to be developed across a number of phases of enquiry (Figure 1), and facilitated further explanation and exploration of unexpected quantitative and qualitative results obtained from patients, staff, direct observations, interviews, and focus groups. Again, this enabled multiple perspectives of reality rather than simplistic acceptance or rejection of a single hypothesis designed prior to collecting any bedside data [39]. Questions were also able to be developed and adapted over time to capture a diversity of views, encompass a variety of sampling methods, and allow for weaknesses associated with one strand to be offset by strengths of the other strand [38,39]. Perhaps most importantly, research questions were designed by clinicians aiming to provide results to inform real world decision-making and improved patient and healthcare outcomes. Hence AR is a method by which the ongoing challenges of practice can be addressed through systematic data gathering and analysis *of* that practice. Such an approach demands a break with research conventions that until now have dominated medical research in favour of a greater understanding of the vagaries of practice and the confidence to move beyond the constraints of research paradigm purity [25,27,39].

Pragmatic research also considers the effectiveness of interventions accounting for varied skills of everyday practitioners, role delineation, shifting accountabilities and work prioritisation, resource limitations, training difficulties, and other workplace cultural and environmental factors that are encountered within routine clinical practice. Results from this study demonstrate, for example, that previously validated nutrition screening tools were not effective in routine clinical practice in response to such differences in staff skills, training, settings and patient populations, leading to substantial patient and healthcare implications [30].

We consider the application of an AR approach to have had an impact on the research setting through the engagement of clinicians *as researchers* in identifying problems and solutions, promoting teamwork, and sustaining outcomes. AR has previously been demonstrated to improve outcomes though improved engagement of isolated team members, changes to multidisciplinary team dynamics, and increased knowledge and awareness of team members regarding the problem under investigation, when compared with research models reliant on less engaged external researchers [37]. To clearly make the point, the process by which interventions have been identified and implemented should be highlighted, as the interventions association with phase IV by themselves are not novel. These factors support the premise that AR itself should be considered an intervention, rather than considering AR processes as confounding outcomes measurement or staining the purity of research quality. For this reason the authors strongly discourage clinicians from simply attempting to duplicate specific interventions demonstrated to be successful in the absence of a pragmatic, AR framework.

It is important to note that subsequent outcomes have demonstrated high patient and clinician relevancy, have high external validity and applicability across settings, and are more likely to answer the question ‘does the intervention work in routine clinical practice' [27]. In short, these clinical *and* research practices fulfill the translational expectations of evidence-based medicine through the processes of evidence-based practice.

### Challenges of multiphase, pragmatic AR approaches

The study design presented in this paper is not without challenges (Table 1). To enable waiving of informed consent and to maximise relevancy to routine clinical practice, outcomes measures are restricted to those routinely available or measured within the context of multidisciplinary clinical care. However, the authors suggest having less, but highly relevant outcomes, is beneficial to those working in the field. Similarly, interventions must be made as part of improvements to routine clinical practice. Inevitably, this limits the opportunities for comparison to before-and-after or across different population groups rather than randomisation. Again, an ideally constructed randomised intervention that has only indirect relevance to clinicians may be less valuable to clinicians than a simple pre-post design that is conducted within the context of routine clinical practice. A multiphase, pragmatic AR approach further allows the flexibility to evaluate the impact of multidisciplinary and multimodal interventions; however, we acknowledge that the opportunities to describe cause and effect or isolate the impact of specific aspects of interventions are limited. The impact of applying an AR process itself must also be taken into account. However this should be considered an asset of AR, rather than a deficit. Multiphase studies require skillful connection of strands over multiple phases and are also recognised as difficult to define as part of the one program. The complex nature of the study design make it more difficult to disseminate results given the conventions of post-positivist studies that tend to favour reductionism by detailing highly selected specific variables and determinism through claims for cause and effect. In addition the proclivity for variable measurement, statistical analysis and reporting (quantitative techniques) may lead to relegation of ‘other’ research as less important or even irrelevant [37,39]. However, there is clearly an increased demand for and interest in studies relevant to clinical practice and this is reflected in changing publication patterns in journals. We would argue that the tenets of pragmatic trials (in this case framed by AR) are an equal partner in the cause of translational research.

## Conclusions

We would conclude by arguing that a multiphase, pragmatic AR approach provides a dynamic framework for clinicians to identify, investigate, and report on multimodal and multidisciplinary interventions conducted within the context of routine clinical practice. Results from this study demonstrated substantially different findings to those previously reported. By minimising selection bias, applying appropriate and clinically relevant diagnostic measures, identifying and introducing flexible interventions in a real world setting, and focusing on outcomes relevant to patients and healthcare providers, outcomes demonstrate high patient and clinician relevancy, have high external validity, and are more likely to answer the question ‘does the intervention work in routine clinical practice'. The authors strongly encourage the development of similar study designs to investigate complex health problems in elderly, multi-morbid patient populations and indeed others where the complexities of comorbidities demand a broader and more flexible research approach.

## Abbreviations

AR: Action research; EBM: Evidence based medicine; PT: Pragmatic trials; RCT: Randomised controlled trial.

## Competing interests

The authors declare they have no competing interests. The lead author is a doctoral candidate investigating barriers and enablers to nutrition care in hip fracture inpatients.

## Authors’ contributions

JJB conceived the study and was the primary contributor to study design, data entry, analysis and interpretation and manuscript drafting and review. TR contributed to study design and manuscript drafting. JDB and SC also contributed to study design and data interpretation. All authors critically revised the manuscript for important intellectual content and have approved the final manuscript.

## Pre-publication history

The pre-publication history for this paper can be accessed here:

http://www.biomedcentral.com/1471-2288/14/98/prepub
